# NEIL3-deficient bone marrow displays decreased hematopoietic capacity and reduced telomere length

**DOI:** 10.1016/j.bbrep.2022.101211

**Published:** 2022-01-18

**Authors:** Tom Rune Karlsen, Maria B. Olsen, Xiang Y. Kong, Kuan Yang, Ana Quiles-Jiménez, Penelope Kroustallaki, Sverre Holm, Glenn Terje Lines, Pål Aukrust, Tonje Skarpengland, Magnar Bjørås, Tuva B. Dahl, Hilde Nilsen, Ida Gregersen, Bente Halvorsen

**Affiliations:** a Research Institute of Internal Medicine, Oslo University Hospital Rikshospitalet, Oslo, Norway; b Institute of Clinical Medicine, Faculty of Medicine, University of Oslo, Oslo, Norway; c Department of Clinical Molecular Biology, University of Oslo and Akershus University Hospital, Lørenskog, Norway; d Simula Research Laboratory, Oslo, Norway; e Section of Clinical Immunology and Infectious Diseases, Oslo University Hospital Rikshospitalet, Oslo, Norway; f Department of Microbiology, Oslo University Hospital Rikshospitalet, Oslo, Norway; g Department of Clinical and Molecular Medicine, Norwegian University of Science and Technology, Trondheim, Norway; h Department of Research and Development, Division of Emergencies and Critical Care, Oslo University Hospital HF, Rikshospitalet, Norway

**Keywords:** NEIL3, Telomeres, Hematopoiesis, Senescence

## Abstract

Deficiency of NEIL3, a DNA repair enzyme, has significant impact on mouse physiology, including vascular biology and gut health, processes related to aging. Leukocyte telomere length (LTL) is suggested as a marker of biological aging, and shortened LTL is associated with increased risk of cardiovascular disease. NEIL3 has been shown to repair DNA damage in telomere regions *in vitro*. Herein, we explored the role of NEIL3 in telomere maintenance *in vivo* by studying bone marrow cells from atherosclerosis-prone NEIL3-deficient mice. We found shortened telomeres and decreased activity of the telomerase enzyme in bone marrow cells derived from *Apoe*^*−/−*^*Neil3*^*−/−*^ as compared to *Apoe*^*−/−*^ mice. Furthermore, *Apoe*^*−/−*^*Neil3*^*−/−*^ mice had decreased leukocyte levels as compared to *Apoe*^*−/−*^ mice, both in bone marrow and in peripheral blood. Finally, RNA sequencing of bone marrow cells from *Apoe*^*−/−*^*Neil3*^*−/−*^ and *Apoe*^*−/−*^ mice revealed different expression levels of genes involved in cell cycle regulation, cellular senescence and telomere protection. This study points to NEIL3 as a telomere-protecting protein in murine bone marrow *in vivo*.

## Highlights

• Deficiency of DNA repair enzyme NEIL3 leads to shortened telomeres in *Apoe*^*−/−*^ mice.
• Hematopoietic capacity is impaired in *Apoe*^*−/−*^ mice lacking NEIL3.
• Transcripts linked to telomere protection are down-regulated in *Apoe*^*−/−*^*/Neil3*^*−/−*^ mice.

## Graphical abstract

**Figure:**
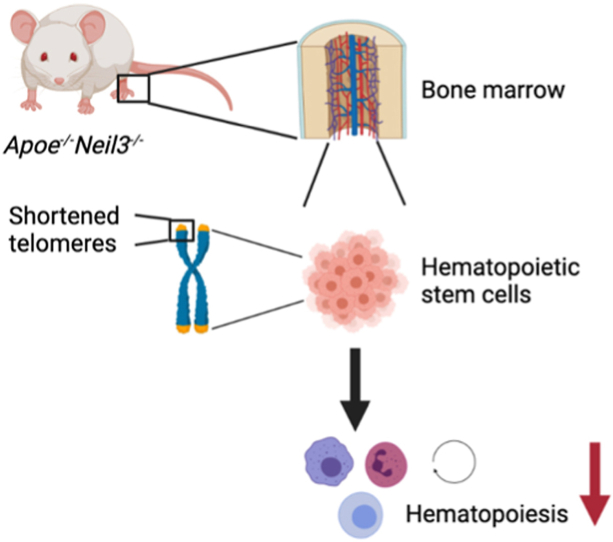

## 1 Introduction

NEIL3 is a DNA glycosylase whose canonical function is DNA repair. Previously, we have shown that single nucleotide polymorphisms in the *NEIL3* gene in a large Norwegian population cohort (HUNT study) was associated with cardiovascular disease (CVD) [1]. Furthermore, we have demonstrated that *Apoe*^*−/−*^*Neil3*^*−/−*^ mice develop more atherosclerosis than *Apoe*^*−/−*^ mice at a mature age (24 weeks) [2,3], but not at a younger age (16 weeks) [4]. Atherosclerosis is associated with premature biological aging [5], a process that is highly interconnected with telomere function [6].

Telomeres are structures composed of repetitive DNA sequences and protein at the end of non-circular chromosomes, protecting genetic information from being lost during cell replication. The DNA replication process leads to gradual shortening of telomeric DNA each cell cycle [7]. Telomeres naturally shorten with age, while accelerated telomere shortening is associated with premature aging [6]. At a critical telomere length limit, the cell undergoes apoptosis or senescence [8]. To circumvent this, cells can express the enzyme telomerase, which elongates telomeres [9]. Telomerase is absent or found at very low levels in adult somatic cells, but is expressed in germ cells, stem cells and cancer cells [10].

Leukocyte telomere length (LTL) is suggested to be a marker of biological aging, and shortened LTL is associated with increased risk of CVD [11]. NEIL3 has been shown to protect telomeres *in vitro* [12]. To our knowledge, this effect of NEIL3 has not yet been demonstrated *in vivo*. We hypothesized that NEIL3 possesses a telomere-protective function also *in vivo*, and that this is linked to the increased atherogenesis we have previously observed in *Apoe*^*−/−*^*Neil3*^*−/−*^ mice [2,3].

## 2 Materials and methods

### 2.1 Animal models

*Neil3*-knockout mice were generated by germline deletion of exons 3–5 as previously described [13]. This was followed by backcrossing into C57BL/6 mice for 10 generations. *Apoe*^*−/−*^*Neil3*^*−/−*^ double knockout mice were generated by crossing *Neil3*^*−/−*^ mice with *Apoe*^*−/−*^ mice (C57BL/6 background), bought from Taconic (Denmark). All mice were born at the Centre for Comparative Medicine, Oslo University Hospital Rikshospitalet, Oslo (Norway). The mice were given chow diet (RM3-P, product code 801700) from Special Diets Services (www.sdsdiets.com) *ad libitum* until sacrifice. Animal experiments were performed in accordance with the European Directive 2010/63/EU. This study has been approved by the Norwegian National Animal Research Authority with license numbers FOTS 22322 and FOTS 8648.

### 2.2 Mouse tissue collection

Blood and bone marrow collection took place at 4 months of age, except for the cell colony forming unit experiment, which took place at 6 months of age. Animals used in the same experiments were age-matched and sacrificed at the same time. Full blood for cell count analysis was collected from the calf vein of the mice, using EDTA-coated capillary tubes. The blood was kept at room temperature and analyzed within 2 h. Bone marrow harvest took place under non-fasting conditions. The mice were anaesthetized with 4% isoflurane in an induction chamber, before application of a mask with 2% isoflurane. *Trans*-thoracic cardiocentesis was performed, and the mice were euthanized by exsanguination. Femurs and tibias were harvested as previously described [14], and muscles were removed from the bones. Bones were rinsed with 70% ethanol and ice-cold HBSS under sterile conditions.

### 2.3 Bone marrow extraction

Bone marrow was extracted from tibias and femurs as previously described [14,15]. Briefly, epiphyses of each bone were cut off to expose the interior of the marrow shaft. Extraction of bone marrow was done by either the flush method [14] or the spin method [15]. For the spin method, the bone was placed in a 200 μL pipette tip inside a 1.5 mL Eppendorf tube, followed by centrifugation for 1 min at 1000 g. For the flush method, bones with cut epiphyses were flushed with 10 mL ice-cold HBSS with a 26-gauge needle. Single-cell suspension was obtained by aspiration through a syringe and passing them through a 100 mm cell strainer (Corning). The cell suspension was centrifuged at 200 g for 5 min at 4 °C and the cell pellet was used for further analyses.

### 2.4 Telomere length analysis by qPCR

Genomic DNA was extracted from bone marrow cell pellets using DNeasy Blood and Tissue kit (Qiagen, Germany) according to the manufacturer's instructions. The bone marrow cells were homogenized using the BeadBeater homogenizer. Average telomere length was measured from total genomic DNA samples using the qPCR method [16] as previously described [17]. The single-copy gene 36B4 was used as reference. Each reaction included 5 μL 2x Power SYBR Green PCR Master mix (Applied Biosystems), 0.1 μL 10 μM telo forward and 0.9 μL 10 μM telo reverse primers [18], 2 μL water and 2 μL genomic DNA (0.5 ng/μL) to yield a 10 μL-reaction. For each single copy gene reaction was included 5 μL 2x Power SYBR Green PCR Master mix (Applied Biosystems), 0.3 μL of 10 μM forward and 0.7 μL 10 μM reverse primers [18], 2 μL water and 2 μL gDNA (5 ng/μl) to yield a 10 μL-reaction. QuantStudio 7 Flex Real-Time PCR System (Applied Biosystems) was used with reaction conditions of 95 °C for 10 min followed by 40 cycles of data collection at 95 °C for 15 s, 60 °C anneal for 30 s, and 72 °C extend for 45 s.

### 2.5 Telomerase activity

Bone marrow cell pellets were lysed in CHAPS buffer (TRAPeze kit, Millipore) for 30 min on ice. Tissues were homogenized using the BeadBeater homogenizer. Protein content determination of lysates was performed using the BCA method with a BSA standard curve. 10 μL of lysate, with concentrations 50–200 ng/μL, was added to a 50 μL extension reaction containing 1x TRAP reaction buffer (10x concentration: 200 mM Tris-HCl, pH 8.3, 15 mM MgCl_2_), 0.4 mg/ml BSA, TS telomerase extension substrate (HPLC purified, 200 nM; 5′-AATCCGTCGAGCAGAGTT-3’), dNTPs (2.5 mM each) and incubated for 40 min at 25 °C, then held at 4 °C. The digital droplet PCR (ddPCR) reaction was assembled containing 1x EvaGreen ddPCR Supermix v2.0 (Bio-Rad), 50 nM TS primer, 50 nM ACX primer, 10 μL of extension product for a final volume of 25 μL. The lysis-extension mixture was subsequently used for the standard ddTRAP protocol, as previously described [19].

### 2.6 Cell colony forming assay

Bone marrow cells were resuspended in PBS and counted. 20 000 cells from each mouse were seeded in RPMI 1640 medium supplemented with 10% FCS and a methylcellulose-based medium with recombinant cytokines for mouse myeloid progenitor cells from StemCell Technologies (Methocult GF M3434, Cat#03434) in a 3 cm Petri dish. The dish was placed inside a 10 cm dish together with a 3 cm dish with water to prevent dehydration. The cells were seeded for 7 days before the colonies were counted manually in a microscope. Counting was performed by two independent researchers, and the average count was used.

### 2.7 Flow cytometry

Bone marrow cells were diluted to 1 mill/mL in flow buffer before erythrocyte lysis (BD FACSLysing Solution, Beckman Dickinson, CA). Cells were incubated with optimally titrated antibodies for 15 min at 4 °C, before analysis using the Milteney MACS Quant. The following antibodies were used: CD45-viogreen, CD115-PE, Ly6G-Pevio770 and CD11b-APC (Milteney biotec). Neutrophils were defined as CD115^-^Ly6G^+^CD11b^+^, eosinophils as CD115^-^Ly6G^−^CD11b^+^, basophiles as CD115^-^Ly6G^−^CD11^dim^ (Supplementary Fig. 1).

### 2.8 Blood cell count

Cell count of total leukocytes, erythrocytes and thrombocytes was determined using an ABX Micros 60 Hematology Analyzer (HORIBA Medical, Japan). 60 μL of full blood sample was transferred to the machine and a standard cell count was performed.

### 2.9 RNA extraction and sequencing

RNA was extracted from bone marrow cell pellets using AllPrep Blood and Tissue kit (Qiagen, Germany) according to the manufacturer's protocol. RNA purity and yield were assessed by spectrophotometer absorbance (Nanodrop ND-1000 Thermo Scientific, Wilmington, DE). The RNA was sent to Novogene Company Limited (UK) for library preparation and sequencing.

### 2.10 Statistics

Statistical analyses were done by Student's *t*-test, using GraphPad Prism version 9. P-values <0.05 were considered significant. Statistical outliers were identified using Grubb's test with *α* = 0.05. For RNA sequencing analyses, the fastp software (v0.20.1) was used to remove contaminated adapters and low-quality reads with phred score below 30 in the pair-end 150 bp raw sequencing files (PMID: 30423086). Salmon (v1.5.2) was used to map the filtered reads to the mouse transcriptome (Gencode Mouse Release M26) with 200 bootstrap iterations (PMID: 28263959, PMID 30357393). To obtain the differentially expressed transcripts (DETs), the Salmon outputs were imported into DESeq2 (v1.32.0) via tximport (v.1.20.0) (PMID: 25516281, PMID 26925227). Transcripts positively correlating to *Neil3-201* expression in *Apoe*^*−/−*^ were uploaded to Metascape for gene functional enrichment analysis (PMID: 30944313). Pearson correlation coefficient was calculated in *Neil3* correlation analysis.

## 3 Results

### 3.1 Shortened telomere length and decreased telomerase activity in *Apoe*^*−/−*^*Neil3*^*−/−*^ mice as compared to *Apoe*^*−/−*^ mice

**Fig. 1:**
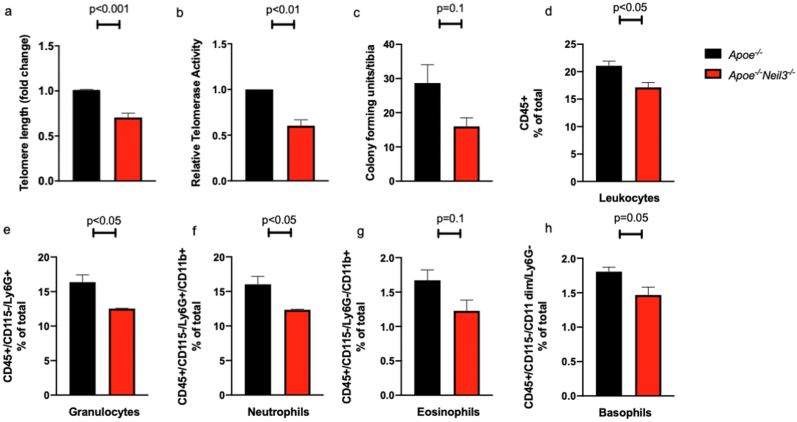
**Decreased telomere length, decreased telomerase activity and decreased cell number in bone marrow cells from *Apoe***^***−/−***^***Neil3***^***−/−***^**mice as compared to *Apoe***^***−/−***^**mice. A)** Telomere length. **B)** Telomerase activity. **C)** Number of colony forming units from bone marrow cells. **D-h**: Levels of leukocytes in bone marrow. **D)** Total leukocytes (CD45^+^). **E)** Total granulocytes (CD45^+^CD115^-^Ly6G^+^). **f)** Neutrophilic granulocytes (CD115^-^Ly6G^+^CD11b^+^). **g)** Eosinophilic granulocytes (CD115^-^Ly6G^−^CD11b^+^). **h)** Basophilic granulocytes (CD115^-^Ly6G^−^CD11^dim^). Results presented as mean ± SEM, n = 3–4.

We have previously shown that *Apoe*^*−/−*^*Neil3*^*−/−*^ mice develop more atherosclerosis than *Apoe*^*−/−*^ mice in an age-dependent manner [4], suggesting a potential role for *Neil3* in accelerated aging that predisposes to enhanced atherogenesis. *In vitro* experiments have shown that NEIL3 maintains and repairs telomeres [12], and we therefore examined if NEIL3 also has similar effects *in vivo*. When comparing telomere length in bone marrow cells, a compartment with high cellular turnover [20], we found that the telomere length was reduced by approximately 30% in *Apoe*^*−/−*^*Neil3*^*−/−*^ mice as compared to *Apoe*^*−/−*^ mice (Fig. 1a). Furthermore, *Apoe*^*−/−*^*Neil3*^*−/−*^ mice had about 40% lower activity of the telomerase enzyme as compared to *Apoe*^*−/−*^ mice (Fig. 1b).

### 3.2 Decreased myelopoiesis in bone marrow of *Apoe*^*−/−*^*Neil3*^*−/−*^mice

Decreased telomere length in bone marrow cells has been linked to decreased hematopoietic capacity [21]. To assess whether this was the case in *Apoe*^*−/−*^*Neil3*^*−/−*^ mice, we performed an *ex vivo* cell colony forming assay of bone marrow cells. There was a trend towards fewer colony forming units from *Apoe*^*−/−*^*Neil3*^*−/−*^ mice as compared to *Apoe*^*−/−*^ mice (Fig. 1c). Furthermore, flow cytometry analysis of white blood cells in the bone marrow of *Apoe*^*−/−*^*Neil3*^*−/−*^ mice and *Apoe*^*−/−*^ mice revealed significantly fewer leukocytes (CD45^+^) and total granulocytes (CD45^+^CD115^-^), including both neutrophils (CD11b^+^Ly6G^+^CD115^-^) and basophils (CD11^dim^Ly6G^−^CD115), in the bone marrow of *Apoe*^*−/−*^*Neil3*^*−/−*^ mice as compared to *Apoe*^*−/−*^mice (Fig. 1d–h).

### 3.3 Decreased peripheral blood cell count in *Apoe*^*−/−*^*Neil3*^*−/−*^mice

**Fig. 2:**
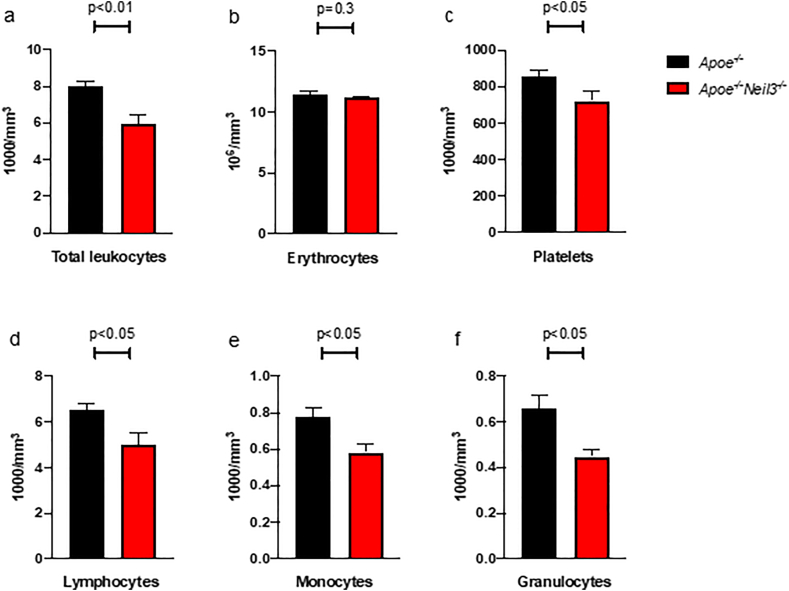
**Decreased peripheral blood leukocytes and platelets in *Apoe***^***−/−***^***Neil3***^***−/−***^**mice as compared to *Apoe***^***−/−***^**mice. a)** Total leukocytes. **b)** Erythrocytes. **c)** Platelets. **d)** Lymphocytes. **e)** Monocytes. **f)** Granulocytes. Results presented as mean ± SEM, n = 6–10.

To investigate the influence of decreased bone marrow cell counts on circulation cells, we next used a cell counter to measure the levels of blood cells in *Apoe*^*−/−*^*Neil3*^*−/−*^mice and *Apoe*^*−/−*^ mice. This revealed a significant decrease in total leukocytes and platelets, but not erythrocytes, in *Apoe*^*−/−*^*Neil3*^*−/−*^ mice as compared to *Apoe*^*−/−*^ mice (Fig. 2a–c). For leukocytes, the decrease was significant in the monocytic, granulocytic, and lymphocytic cell lines (Fig. 2d–f). This supports an impaired hematopoietic function in *Apoe*^*−/−*^*Neil3*^*−/−*^ mice.

### 3.4 RNA sequencing data support a role for reduced telomere maintenance in impaired hematopoiesis

**Fig. 3:**
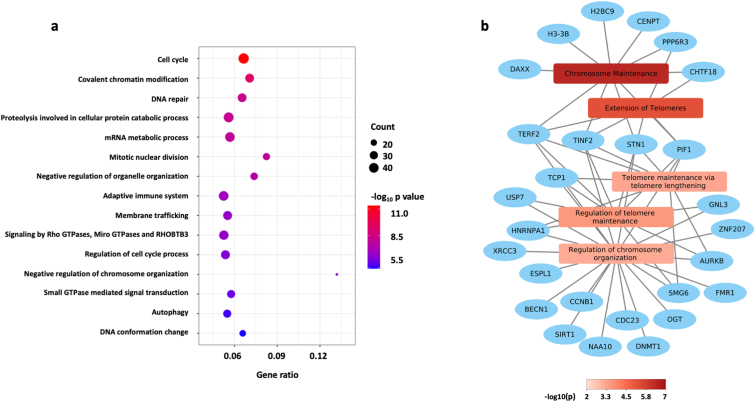
**Genes involved in cell division, maturation and telomere protection are co-expressed with NEIL3. a)** Top gene ontology (GO) terms found for genes co-expressed with *Neil3*. Gene enrichment analysis including transcripts that positively correlate with NEIL3-expression in *Apoe*^*−/−*^ mice. Count: the number of enriched genes in the corresponding GO term. Gene Ratio: number of enriched genes relative to total genes in the corresponding GO term. **b)** Network plot showing significantly enriched terms and pathways related to telomere maintenance. n = 4.

**Fig. 4:**
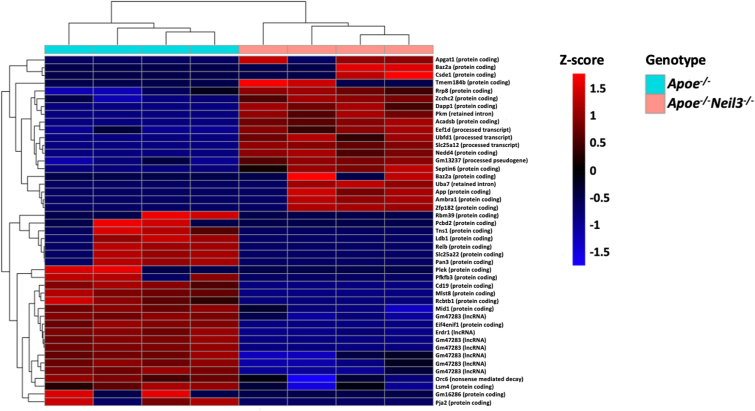
**RNA sequencing reveals key transcriptional differences in bone marrow cells from *Apoe***^***−/−***^***Neil3***^***−/−***^**mice as compared to *Apoe***^***−/−***^**mice.** Heatmap showing analysis of regulated transcripts in *Apoe*^*−/−*^*Neil3*^*−/−*^ mice as compared to *Apoe*^*−/−*^ mice. Columns represent individual mice (light blue: *Apoe*^*−/−*^, pink: *Apoe*^*−/−*^*Neil3*^*−/−*^), while rows represent different transcripts. Colors represent the z-score of the transcript in question. n = 4. (For interpretation of the references to color in this figure legend, the reader is referred to the Web version of this article.)

To better understand the mechanisms underlying the reduced hematopoiesis in *Apoe*^*−/−*^*Neil3*^*−/−*^ mice, we performed RNA sequencing of bone marrow cells from *Apoe*^*−/−*^*Neil3*^*−/−*^ mice and *Apoe*^*−/−*^ mice. First, we performed a correlation analysis to find transcripts that correlate with *Neil3* expression in *Apoe*^*−/−*^ mice. We found that 713 transcripts positively correlated (p < 0.005, correlation coefficient >0.86) to *Neil3* transcript levels. To examine their possible biological roles, gene enrichment analysis was performed. The top gene ontology (GO) terms and pathways included *Cell cycle, Covalent chromatin modification* and *DNA repair*; highly relevant for cell maturation and cell division (Fig. 3a). Furthermore, the regulated GO term *Negative regulation of chromosome organization* (Fig. 3a) shares many common genes with significantly enriched terms and pathways related to telomere maintenance, as presented in a Network plot (Fig. 3b). Next, transcript levels from *Apoe*^*−/−*^*Neil3*^*−/−*^ derived bone marrow cells were compared to cells from *Apoe*^*−/−*^ mice, detecting 45 differently expressed transcripts (Fig. 4). Several of the regulated transcripts in *Apoe*^*−/−*^*Neil3*^*−/−*^ cells are involved in cell division, including *Zcchc2* and *mlst8*, and in telomere protection, such as *Erdr1*, *Mid1* and *Lsm4*. In addition, several transcriptional variants of the gene *Gm47283*, which shares significant homology with *Erdr1* [22,23], were strongly down-regulated in *Apoe*^*−/−*^*Neil3*^*−/−*^ cells. Finally, several markers of cellular senescence, such as *Acadsb*, *Baz2a* and *Csde1* were upregulated in *Apoe*^*−/−*^*Neil3*^*−/−*^ cells as compared to *Apoe*^*−/−*^ cells.

## 4 Discussion

In this study we show that *Apoe*^*−/−*^*Neil3*^*−/−*^ mice have shortened telomeres in their bone marrow cells as compared to *Apoe*^*−/−*^ mice, accompanied by decreased hematopoiesis with fewer mature peripheral blood cells. In addition, we show that several genes related to the cell cycle, maturation of cells and telomere maintenance are downregulated in *Apoe*^*−/−*^*Neil3*^*−/−*^ bone marrow cells. Our findings may suggest a link between reduced telomere maintenance, impaired hematopoiesis and the previously reported increased atherogenesis in *Apoe*^*−/−*^*Neil3*^*−/−*^ mice.

Telomeres shorten with age and accelerated telomere shortening is associated with premature aging [6,8]. In agreement with our current results, shortening of telomeres has also been linked to bone marrow failure [21]. Furthermore, leukocyte telomere length has been suggested as a marker of biological aging, and is associated with increased risk of CVD [11,24,25], which is in line with our previous reports of increased atherosclerosis in old, but not young *Apoe*^*−/−*^*Neil3*^*−/−*^ mice [[2], [3], [4]]. Whether there is a causal link between leukocyte telomere length and increased atherosclerosis in *Apoe*^*−/−*^*Neil3*^*−/−*^ mice, however, remains to be determined. One mechanistic possibility is that the decreased telomere length in leukocytes extends to other tissues, as suggested by others [26,27], leading to cellular senescence [6]. Senescence has been linked to atherogenic properties in cells such as endothelial cells [28] and vascular smooth muscle cells [29]. Interestingly, senescent macrophages have been shown to promote atherosclerosis through impaired cholesterol efflux [30,31], which is in agreement with our previous report of decreased cholesterol efflux in macrophages derived from *Apoe*^*−/−*^*Neil3*^*−/−*^ mice [3]. In the current study, several markers of cellular senescence, such as *Acadsb* [32], *Baz2a* [33] and *Csde1* [34] were upregulated in *Apoe*^*−/−*^*Neil3*^*−/−*^ cells as compared to *Apoe*^*−/−*^ cells, suggesting a process of beginning or progressing senescence in these cells. Notably, NEIL3 has recently been reported to defend against cellular senescence in human liver cells by protecting telomeres against oxidative damage [35], strengthening the validity of our results.

RNA sequencing also revealed several other interesting differences between *Apoe*^*−/−*^*Neil3*^*−/−*^ and *Apoe*^*−/−*^ bone marrow cells. Of note, *Lsm4*, which has been shown to maintain telomere length by associating with the telomerase enzyme [36], was significantly downregulated in *Apoe*^*−/−*^*Neil3*^*−/−*^ cells as compared to *Apoe*^*−/−*^ cells. Also, the transcripts *Erdr1* and *Mid1*, as well as several transcriptional variants of the gene *Gm47283*, an *Erdr1* homolog [22], were downregulated in *Apoe*^*−/−*^*Neil3*^*−/−*^ cells. *Mid1* and *Erdr1* are parts of the pseudoautosomal region of the X chromosome [37]. Transcription of these genes is linked to expression of what is known as *Telomeric Repeat Containing RNAs* (TERRA) [37], which have been associated with telomere protection [38] and activation of telomerase [39]. Downregulation of these genes in *Apoe*^*−/−*^*Neil3*^*−/−*^ bone marrow cells may therefore suggest loss of protective mechanisms, causing telomere shortening and decreased telomerase activity, as observed in the *Apoe*^*−/−*^*Neil3*^*−/−*^ mouse model.

Moreover, transcripts relevant for cell division were regulated in *Apoe*^*−/−*^*Neil3*^*−/−*^ cells, including upregulation of *Zcchc2*, which is reported to suppress tumorigenesis [40], and downregulation of *Mlst8*, deletion of which has been found to decrease tumor cell proliferation [41]. Together, these data suggest that NEIL3 acts in concert with genes involved in important protective processes during cell division and aging, and that loss of NEIL3 causes dysregulated gene expression, resulting in disruption of cell division regulation.

This study has some limitations. The number of mice were low, especially in the bone marrow cell count analyses, which may also be reflected in some of the results not reaching statistical significance. Furthermore, we have not determined a direct mechanistic interaction between NEIL3 and telomeres or telomere-protecting molecules.

In conclusion, this study shows that loss of NEIL3 causes reduced telomere length in murine *Apoe*^*−/−*^ bone marrow cells, accompanied by decreased leukocyte counts in bone marrow and in peripheral circulation. We hypothesize that NEIL3 functions as a telomere-protecting protein in bone marrow and that loss of NEIL3 leads to increased risk of age-related disease such as atherosclerosis. The causal relationship, however, needs further investigation.

## Declaration of competing interest

The authors declare that they have no known competing financial interests or personal relationships that could have appeared to influence the work reported in this paper.

## Appendix A Supplementary data

Supplemental Figure 1 Gating strategy for characterization of leukocytes. CD45^+^ cells were counted as leukocytes, neutrophils were defined as CD115^-^Ly6G^+^CD11b^+^, eosinophils were defined as CD115^-^Ly6G^−^CD11b^+^ and basophils as CD115^-^Ly6G^−^CD11^dim^. Supplemental Figure 1
