# Supplementary figures and images for: NEIL3-deficient bone marrow displays decreased hematopoietic capacity and reduced telomere length
Source: Biochem Biophys Rep. 2022 Jan 18;29:101211. doi: 10.1016/j.bbrep.2022.101211 (PMC8777121; doi:10.1016/j.bbrep.2022.101211)

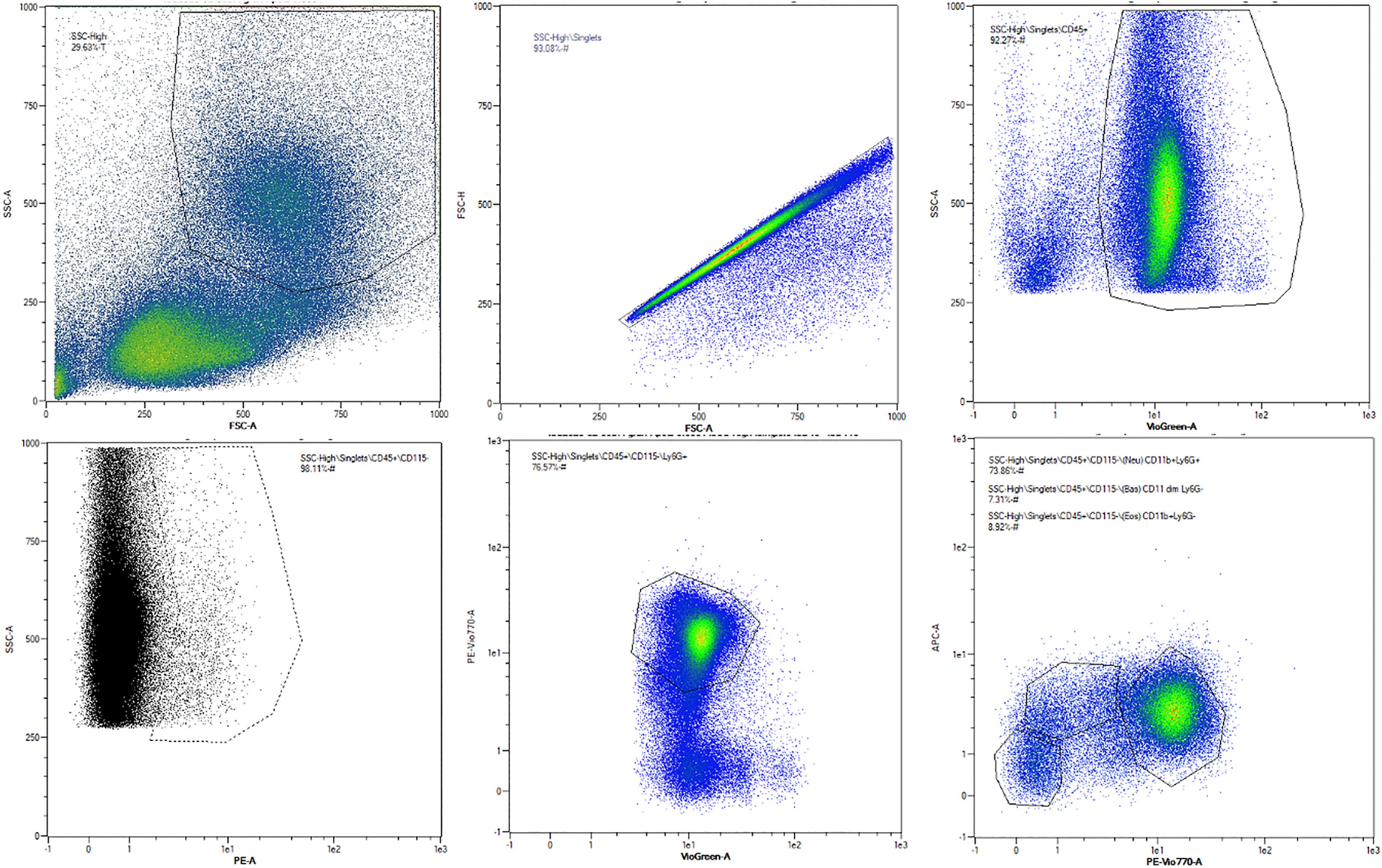

Supplement: Supplemental Figure 1 — Gating strategy for characterization of leukocytes. CD45+ cells were counted as leukocytes, neutrophils were defined as CD115-Ly6G+CD11b+, eosinophils were defined as CD115-Ly6G−CD11b+ and basophils as CD115-Ly6G−CD11dim. [file mmcfigs1.jpg]
